# The relapsing fever spirochete *Borrelia turicatae* persists in the highly oxidative environment of its soft‐bodied tick vector

**DOI:** 10.1111/cmi.12987

**Published:** 2019-01-04

**Authors:** Travis J. Bourret, William K. Boyle, Amanda K. Zalud, Jesus G. Valenzuela, Fabiano Oliveira, Job E. Lopez

**Affiliations:** ^1^ Department of Medical Microbiology and Immunology Creighton University Omaha Nebraska; ^2^ Laboratory of Malaria and Vector Research National Institute of Allergy and Infectious Diseases, National Institutes of Health Rockville Maryland; ^3^ Departments of Pediatrics and Molecular Virology and Microbiology Baylor College of Medicine Houston Texas

## Abstract

The relapsing fever spirochete *Borrelia turicatae* possesses a complex life cycle in its soft‐bodied tick vector, *Ornithodoros turicata*. Spirochetes enter the tick midgut during a blood meal, and, during the following weeks, spirochetes disseminate throughout *O. turicata*. A population persists in the salivary glands allowing for rapid transmission to the mammalian hosts during tick feeding. Little is known about the physiological environment within the salivary glands acini in which *B. turicatae* persists. In this study, we examined the salivary gland transcriptome of *O. turicata* ticks and detected the expression of 57 genes involved in oxidant metabolism or antioxidant defences. We confirmed the expression of five of the most highly expressed genes, including glutathione peroxidase (*gpx*), thioredoxin peroxidase (*tpx*), manganese superoxide dismutase (*sod‐1*), copper‐zinc superoxide dismutase (*sod‐2*), and catalase (*cat*) by reverse‐transcriptase droplet digital polymerase chain reaction (RT‐ddPCR). We also found distinct differences in the expression of these genes when comparing the salivary glands and midguts of unfed *O. turicata* ticks. Our results indicate that the salivary glands of unfed *O. turicata* nymphs are highly oxidative environments where reactive oxygen species (ROS) predominate, whereas midgut tissues comprise a primarily nitrosative environment where nitric oxide synthase is highly expressed. Additionally, *B. turicatae* was found to be hyperresistant to ROS compared with the Lyme disease spirochete *Borrelia burgdorferi*, suggesting it is uniquely adapted to the highly oxidative environment of *O. turicata* salivary gland acini.

## 1 INTRODUCTION

The relapsing fever (RF) spirochete *Borrelia turicatae* is endemic in the southern United States and Latin America and is transmitted by *Ornithodoros turicata* (Davis, 1943). During blood feeding on an infected mammal, *B. turicatae* enters the midgut of *O. turicata*, and within weeks, disseminates to various tick organs including the salivary glands, synganglion, and reproductive tissues (Davis, 1941). *O. turicata* goes through at least five nymphal stages prior to becoming adults, during which the spirochetes are transstadially maintained (Krishnavajhala et al., 2017). *B. turicatae* can also survive prolonged starvation periods of at least five years within *O. turicata* (Davis, 1943). Moreover, prior studies indicated that *B. turicatae* resides within the lumen of salivary gland acini, facilitating the pathogen's rapid transmission to naïve mammalian hosts (Boyle, Wilder, Lawrence, & Lopez, 2014; Krishnavajhala et al., 2017).

Little is known about the environmental stresses *B. turicatae* encounters during *O. turicata* colonisation. Presumably, *B. turicatae* faces shifts in temperature, pH, nutrient availability, osmolarity, and host‐derived reactive oxygen species (ROS) and reactive nitrogen species (RNS), which all significantly depend on whether *O. turicata* has recently fed or is within a period of prolonged starvation. For example, a study on the related soft‐bodied tick *Ornithodoros moubata*, revealed that the midgut pH can rise to as high as 7.6 during the blood meal and be reduced to pH 6.4–6.8 during periods of starvation (Grandjean, 1983). In contrast, the pH of *Ornithodoros* saliva is unknown. Previously, we showed that *Ixodes scapularis*, the hard‐bodied tick vector of the Lyme disease (LD) spirochete *Borrelia burgdorferi*, produced RNS in both its salivary glands and midguts (Bourret et al., 2016). We have found ROS are generated in these tissues as well (data not shown). ROS and RNS appear to be substantial environmental challenges for *B. burgdorferi* during both blood meal acquisition and during starvation in *I. scapularis* nymphs, as ROS‐sensitive and RNS‐sensitive *B. burgdorferi* strains harbouring mutations in antioxidant defence genes show poor survival compared with their wild‐type counterparts (Bourret et al., 2016; Eggers et al., 2011; Li et al., 2007). To the best of our knowledge, the production of ROS and RNS in *Ornithodoros* ticks has not been reported; however, transcriptomic and proteomic studies have identified a variety of oxidant metabolism and antioxidant defence genes expressed in soft‐bodied ticks that transmit RF *Borrelia* (Francischetti, Mans, et al., 2008; Francischetti, Meng, et al., 2008; Landulfo et al., 2017; Oleaga, Obolo‐Mvoulouga, Manzano‐Roman, & Perez‐Sanchez, 2015; Oleaga, Obolo‐Mvoulouga, Manzano‐Roman, & Perez‐Sanchez, 2017b; Oleaga, Obolo‐Mvoulouga, Manzano‐Roman, & Perez‐Sanchez, 2017a).

In the following study, we used RNAseq to determine the transcriptome of *O. turicata* salivary glands to gain insights into the pressures *B. turicatae* encounters during colonisation of its tick vector. On the basis of these results, we examined the expression of genes involved in tick oxidant metabolism and antioxidant defences, along with the production of ROS and RNS in *O. turicata* salivary glands and midguts. This indicated *O. turicata* salivary glands are highly oxidative environments compared with the tick midguts. Accordingly, we found that *B. turicatae* was highly resistant to killing by H_2_O_2_ when compared with the LD spirochete *B. burgdorferi*, suggesting the pathogen is uniquely adapted to the oxidative environment of the salivary gland.

## 2 RESULTS

### 2.1 Expression of antioxidant defence genes in unfed *O. turicata* nymphs

We recently reported *B. turicatae* is localised within the acini lumen of salivary glands from unfed *O. turicata* nymphs (Krishnavajhala et al., 2017), which likely contributes to its ability to be rapidly transmitted from its tick vector to a naïve mammalian host. To further understand the microenvironment of the *O. turicata* acini lumen and how it may affect *B. turicatae* gene expression, physiology, and overall virulence, we assessed the salivary gland transcriptome of uninfected *O. turicata* nymphs by RNAseq. Using the HiSeq2500 sequencer, a total of 1.51 × 10^7^ single‐end reads were generated averaging 252 bp in length. The reads were assembled into 10,990 contigs that were annotated. Of the 10,984 contigs, 6,755 had greater than 75% coverage to their best match in the National Center for Biotechnology Information (NCBI) nonredundant database, and 8,183 had e‐values by blastp of less than 1 × 10^−15^. The families of proteins are shown in Table S1, and this Transcriptome Shotgun Assembly project was deposited at the DDBJ/EMBL/GenBank under the accession GCJJ01000001‐GCJJ010065871. Several antioxidant defence genes were among the most highly expressed genes in the RNAseq analyses, including a glutathione peroxidase and a thioredoxin peroxidase (Table 1). Fifty‐five additional genes involved in either antioxidant defence or the generation of ROS and RNS were detected.

**Table 1:** Select *Ornithodoros turicata* genes expressed in the salivary glands involved in oxidant metabolism or antioxidant defences

Name	Number of reads	RPKM	Putative gene function
Otc‐12454	6,751	1249.7	Glutathione peroxidase partial (*gpx*)*
Otc‐12452	5,787	1231.9	Glutathione peroxidase partial
Otc‐70453	5,608	365.5	Thioredoxin peroxidase (*tpx*)*
Otc‐70741	2,769	157.9	Manganese superoxide dismutase (*sod‐1*)*
OtcSigP‐44241	641	135.0	Superoxide dismutase Cu‐Zn–signalP detected
OtcSigP‐45577	637	134.1	Superoxide dismutase Cu‐Zn–signalP detected
Otc‐69486	1,520	125.3	Amby‐am‐2533 superoxide dismutase cu‐zn (*sod‐2*)*
Otc‐67400	859	116.7	Selenoprotein K
Otc‐73596	1,053	92.8	Selenoprotein t partial–2 predicted membrane helices
OtcSigP‐2498	554	85.9	Thioredoxin peroxidase–signalP detected
Otc‐60505	3,148	80.1	Catalase pediculus us corporis catalase (*cat*)*
Otc‐42362	4,402	50.8	Nitric oxide synthase (*nos*)*
Otc‐73293	1,156	9.6	Dual oxidase–7 predicted membrane helices (*duox1*)*
OtcSigP‐25713	353	9.0	Dual oxidase–7 predicted membrane helices (*duox2*)*

* genes selected for analysis by RT‐ddPCR.

**Figure 1:**
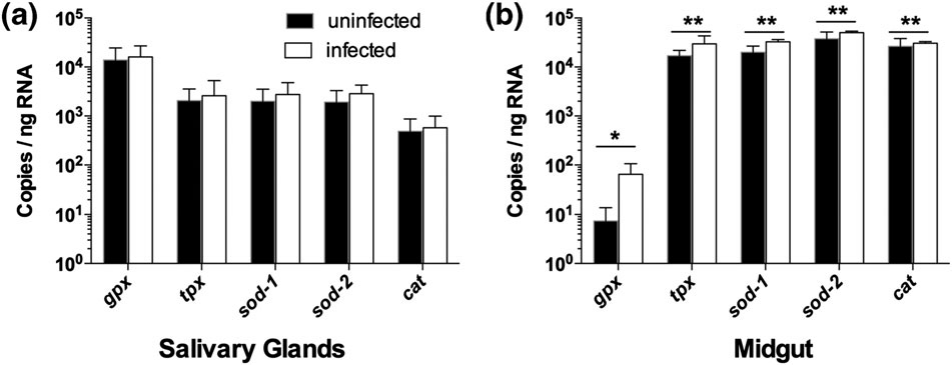
Antioxidant gene expression in *Ornithodoros turicatae* salivary glands and midguts. The expression of antioxidant defence genes were compared by RT‐ddPCR in the salivary glands (a) and midguts (b) of uninfected and *Borrelia turicatae*‐infected *O. turicata* nymphs. The number of copies of each gene were normalised to the quantity of RNA (ng) subjected to reverse transcription. Data represent the mean ± *SD* of three biological replicates and statistical significance was determined using a two‐tailed Student's *t*‐test. No statistically significant differences in gene expression were observed comparing uninfected with infected samples. *p < 0.005 for higher expression in salivary glands compared with midguts. **p < 0.005 for lower expression in salivary glands compared with midguts

Expression patterns for a subset of these genes was confirmed by reverse‐transcriptase droplet digital polymerase chain reaction (RT‐ddPCR) in both uninfected and *B. turicatae*‐infected *O. turicata* nymphs (Figure 1a). The genes included glutathione peroxidase (*gpx*), thioredoxin peroxidase (*tpx*), an Mn‐dependent superoxide dismutase (*sod‐1*), a Cu^2+^/Zn^2+^ superoxide dismutase (*sod‐2*), and a catalase (*cat*). Although the infection status of *O. turicata* nymphs was not associated with differences in the expression of these selected antioxidant defence genes, we did observe significant differences in the expression of these genes when comparing their expression in salivary glands and midguts (Figure 1a,b). The expression of *gpx* was approximately 50‐fold higher in the salivary glands compared with the midgut, whereas the expression levels of *tpx*, *sod‐1*, *sod‐2*, and *cat* were approximately 10–30‐fold lower in the salivary glands compared with the midguts. The observed differences in the expression of antioxidant genes in the salivary glands and midguts of *O. turicata* nymphs suggested the microenvironments of the salivary gland acini and the midgut lumen may present varying degrees of oxidative and nitrosative stresses that *B. turicatae* must endure during infection of its tick vector.

### 2.2 Expression of genes involved in ROS and RNS production

**Figure 2:**
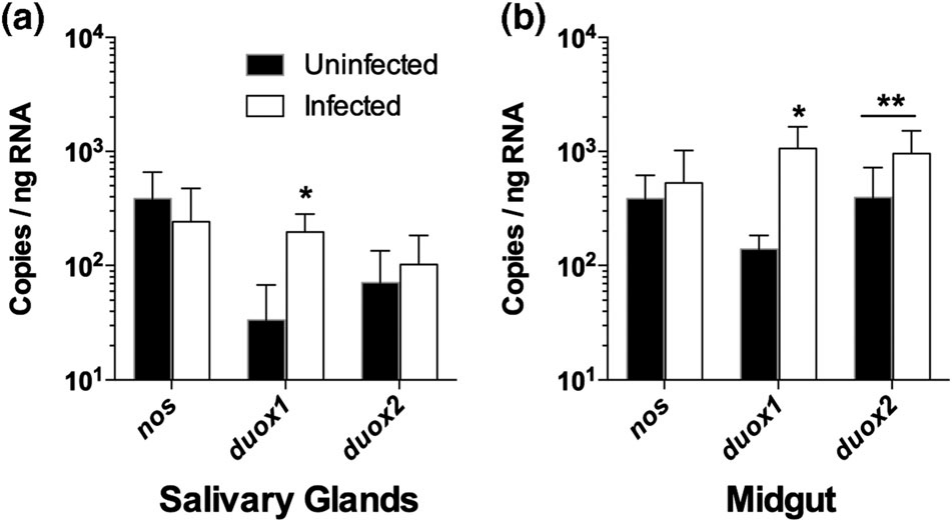
Expression of genes encoding reactive oxygen species (ROS) and reactive nitrogen species‐producing enzymes (RNS). The expression of genes involved in ROS and RNS production were compared by RT‐ddPCR in the salivary glands (a) and midguts (b) of uninfected and *Borrelia turicatae*‐infected *Ornithodoros turicata* nymphs. The number of copies of each gene were normalised to the quantity of RNA (ng) subjected to reverse transcription. Data represent the mean ± *SD* of three biological replicates and statistical significance was determined using a two‐tailed Student's *t*‐test.*p < 0.05 compared with uninfected controls. **p < 0.05 for lower expression in salivary glands compared with midguts

Previous studies examining the hard‐bodied tick *I. scapularis* have implicated dual oxidase (*duox*) and nitric oxide synthase (*nos*) as the primary sources of ROS and RNS production in the tick midgut during infection (Bourret et al., 2016; Yang, Smith, Williams, & Pal, 2014). Our RNAseq analyses indicate that two putative *duox* genes (*duox1* and *duox2*) and a single *nos* gene are expressed in *O. turicata* salivary glands (Table 1). The expression of each of these genes was confirmed by RT‐ddPCR (Figure 2). Although the infection status of *O. turicata* ticks does not have an impact on the expression of *nos* or *duox2* in either salivary glands or midguts, we did observe an increase in the expression of *duox1* in both tissue types in *B. turicatae*‐infected nymphs. Additionally, *duox2* was expressed at higher levels in *O. turicata* midguts compared with the salivary glands. Collectively, these data support the hypothesis that both the *O. turicata* salivary glands and midguts are sites of ROS and RNS production.

**Figure 3:**
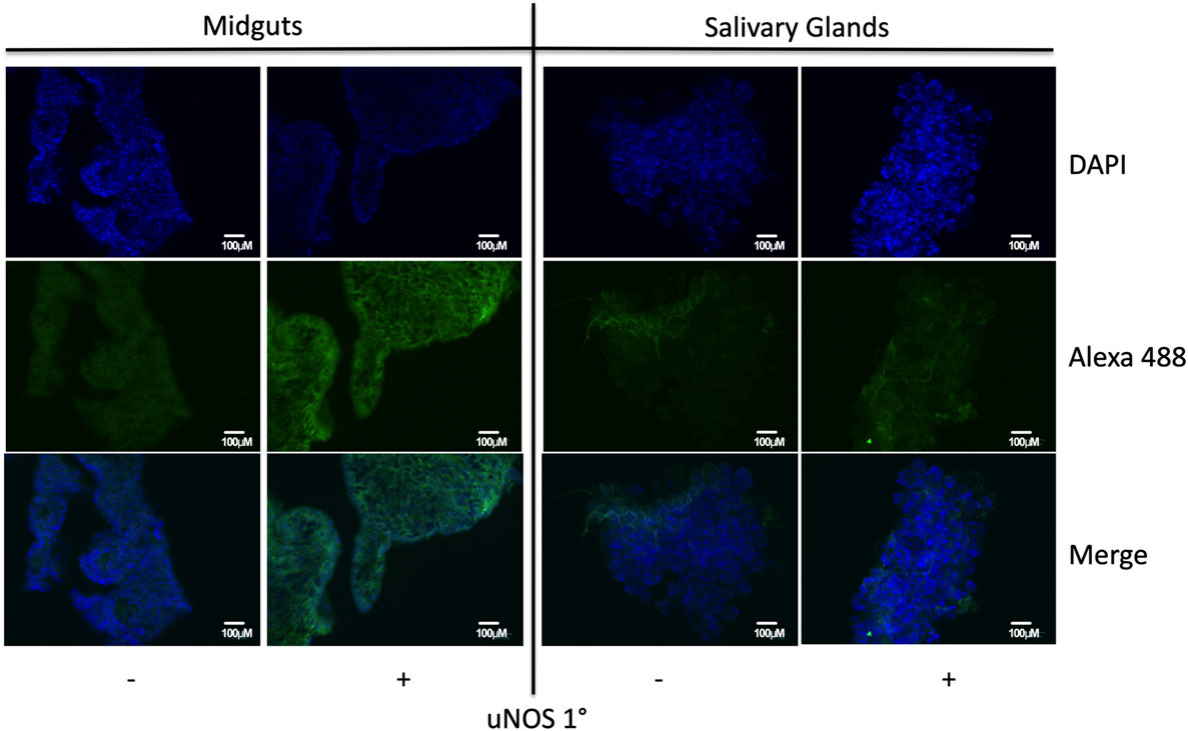
Expression of NOS in *Ornithodoros turicata* salivary glands and midguts. Salivary glands and midguts dissected from *O. turicata* nymphs were incubated in rabbit serum (−) or a universal NOS antibody (+), followed by incubation with an Alexa Fluor 488 secondary antibody and DAPI. Tissues were visualised by fluorescent microscopy using the blue channel (Ex_λ_ = 355 nm, Em_λ_ = 433 nm) and the green channel (Ex_λ_ = 480 nm, Em_λ_ = 517 nm) of the ZOE Flourescent Cell Imager (Bio‐Rad)

Previous work indicated NOS is expressed in both the salivary glands and midguts of the hard‐bodied tick *I. scapularis* by immunofluorescence using a universal NOS antibody (Bourret et al., 2016). In contrast to *I. scapularis*, NOS expression appears to be highest in the midguts of *O. turicata*, whereas the salivary glands show very little expression of NOS (Figure 3). This data, coupled with our RNAseq and RT‐ddPCR data (Table 1 & Figure 3) suggest RNS production in the midgut promotes a nitrosative environment in unfed *O. turicata* ticks.

### 2.3 Production of ROS and RNS in unfed *O. turicata* nymphs

**Figure 4:**
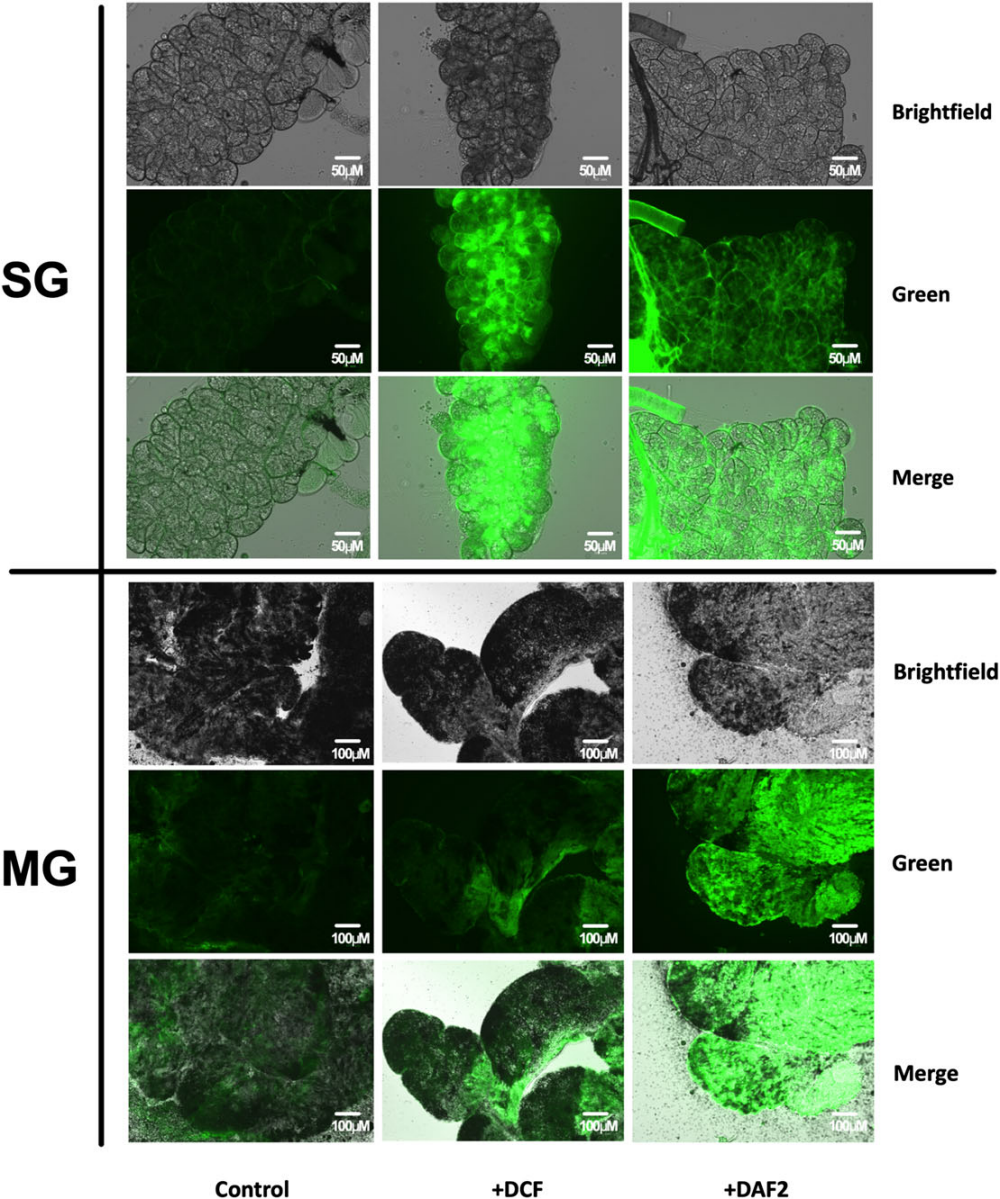
Generation of reactive oxygen species (ROS) and reactive nitrogen species (RNS) in *Ornithodoros turicata* salivary glands and midguts. The presence of ROS and RNS in salivary glands and midguts dissected from *O. turicata* nymphs was compared by incubating tissues for 10 min in the presence or absence of the 25 μM DCF‐DA or 25 μM DAF2‐DA. Brightfield images and Green Channel (Ex_λ_ = 480 nm, Em_λ_ = 517 nm) images were captured for tissues collected from three individual ticks per treatment. The images represent those collected from one tick per treatment

Our transcriptional data, coupled with immunofluorescence data, suggest the salivary glands and midguts of unfed *O. turicata* ticks are highly oxidative and nitrosative environments, which may be substantial hurdles for *B. turicatae* infection of these tissues. Therefore, we probed salivary glands and midguts dissected from unfed *O. turicata* nymphs for ROS and RNS production using the fluorogenic substrates 2,7‐dichloroflurescein diacetate (DCF‐DA) and 4,5‐diaminofluorescein diacetate (DAF2‐DA). DCF‐DA is a commonly used indicator of ROS, including the powerful oxidant peroxynitrite (ONOO^−^), which is generated from the reaction of superoxide (O_2_^−^) and NO (Kalyanaraman et al., 2012). We observed robust staining for ROS in the salivary glands of *O. turicata* nymphs and markedly less staining in the midguts (Figure 4). DAF2‐DA is commonly used as a probe for NO production, but is likely nitrosated by dinitrogen trioxide (N_2_O_3_) following the autooxidation of NO by O_2_ to produce the highly fluorescent product triazolofluorescein (DAF‐2 T) (Espey, Miranda, Thomas, & Wink, 2001). We observed robust fluorescence in both salivary glands and midguts probed with DAF2‐DA, suggesting NO was being produced in both tissues. The detection of NO in the *O. turicata* midgut is consistent with the high levels of *nos* transcription (Table 1 & Figure 2) and NOS protein expression detected by immunofluorescence (Figure 3). Together, these data indicate the salivary glands are sites of robust production of O_2_^−^ and NO in unfed *O. turicata* nymphs, leading to a highly oxidative environment, whereas the midgut appears to be primarily a nitrosative environment where NO production from NOS is elevated compared with O_2_^−^ production by DUOX enzymes.

### 2.4 *B. turicatae* and *B. burgdorferi* exhibit disparate sensitivities to ROS and RNS produced in vitro

**Figure 5:**
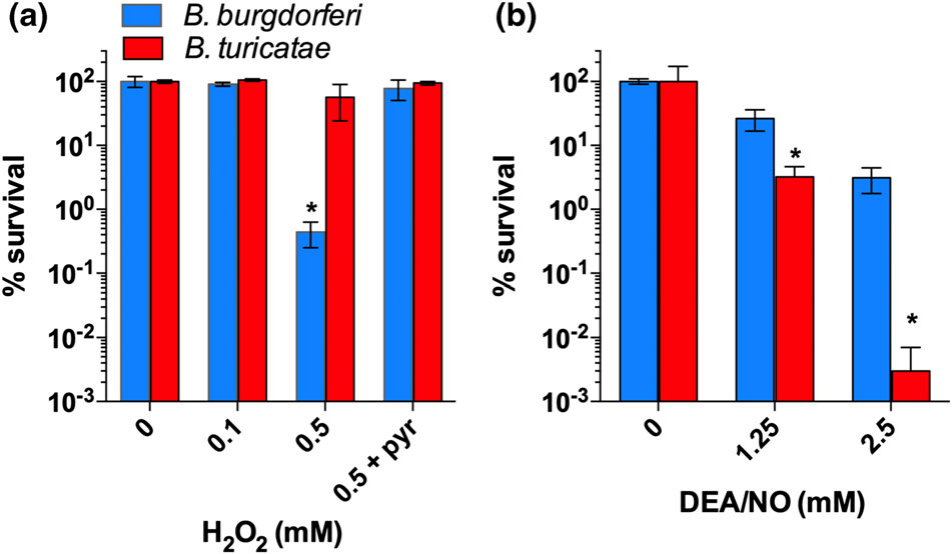
Disparate sensitivities of *Borrelia turicatae* and *Borrelia burgdorferi* to reactive oxygen species and reactive nitrogen species (ROS) and (RNS). *B. turicatae* and *B. burgdorferi* strains grown to a cell density of ~5 × 10^7^ cells ml^−1^ in mBSK medium were exposed to increasing concentrations of H_2_O_2_ (a) or the NO donor diethylamine NONOate (DEA/NO) for 2 h under microaerobic conditions (5% CO_2_, 3% O_2_) at 34°C. Sodium pyruvate (pyr) was added to selected cultures as a scavenger of H_2_O_2_. Following treatment, serial dilutions of spirochetes were prepared in mBSK and plated on solid mBSK media. Data represent the mean % survival ± *SD* of four to five biological replicates. Statistical analysis was performed using a two‐way ANOVA with a Tukey's multiple comparisons test. *p < 0.01 compared with untreated controls

Previously, we have shown *B. turicatae* colonises and persists in both the salivary glands and midguts of *O. turicata* ticks (Boyle et al., 2014; Krishnavajhala et al., 2017). Our data indicate *B. turicatae* must withstand both oxidative and nitrosative environments while it resides in the midguts and salivary glands of *O. turicata* ticks. Several studies have explored the susceptibility of the LD spirochete *B. burgdorferi* to killing by both ROS and RNS (Bourret et al., 2016; Bourret, Boylan, Lawrence, & Gherardini, 2011; Boylan et al., 2006; Boylan, Lawrence, Downey, & Gherardini, 2008; Esteve‐Gassent, Elliott, & Seshu, 2009; Hyde, Shaw, Smith III, Trzeciakowski, & Skare, 2009; Troxell et al., 2014; Troxell, Xu, & Yang, 2012; Wang, Lutton, Olesik, Vali, & Li, 2012). Moreover, a variety of genes have been implicated in the resistance of *B. burgdorferi* ROS and RNS produced during infection of both its tick vector and various mammalian hosts (Bourret et al., 2016; Eggers et al., 2011; Li et al., 2007). To date, there are no published studies examining the susceptibility of relapsing fever spirochetes to oxidative or nitrosative stresses. Therefore, we compared the susceptibility of *B. turicatae* and *B. burgdorferi* with killing by H_2_O_2_ and the NO donor diethylamine NONOate (DEA/NO) (Figure 5). Greater than 99% of *B. burgdorferi* were killed following exposure to 0.5 mM H_2_O_2_, whereas *B. turicatae* viability was unaffected (Figure 5a). In accord with previously published studies (Bourret et al., 2016; Troxell et al., 2014), the addition of 7 mM sodium pyruvate to the BSK II culture media rescued *B. burgdorferi* from killing by H_2_O_2_, which is presumably due to the ability of pyruvate to efficiently scavenge H_2_O_2_. In contrast, *B. turicatae* showed increased susceptibility to killing following exposure to 1.25 mM and 2.5 mM DEA/NO compared with *B. burgdorferi* (Figure 5b). These data suggest *B. turicatae* is more resistant to oxidative stress compared with *B. burgdorferi*, whereas *B. burgdorferi* is more adept at resisting nitrosative stress than *B. turicatae*.

### 2.5 Expression of putative antioxidant defence genes in *B. burgdorferi* and *B. turicatae*

**Figure 6:**
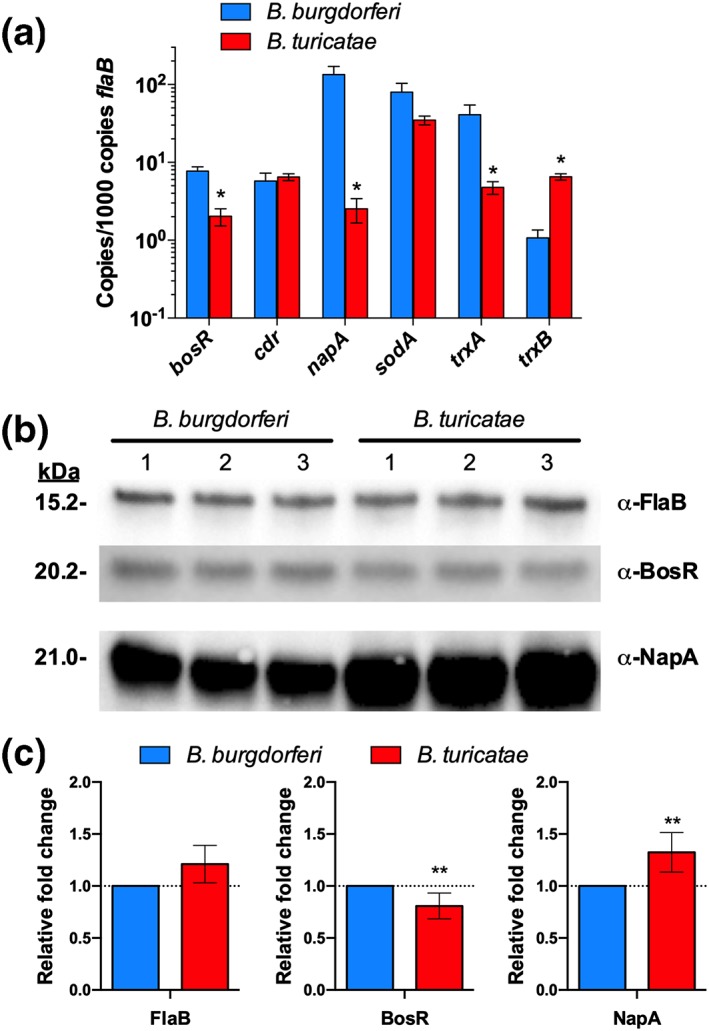
Expression of antioxidant defence genes in *Borrelia turicatae* and *Borrelia burgdorferi*. The expression of putative antioxidant defence genes was compared by RT‐qPCR using RNA harvested from cultures of *B. turicatae* and *B. burgdorferi* grown in mBSK under microaerobic conditions to a cell density of ~5 × 10^7^ cells ml^−1^ using flagellin (*flaB*) as a reference gene (a). Data represent the mean ± *SD* of three biological replicates. Statistical analysis was performed using a two‐tailed, students *t*‐test. *P < 0.01 comparing the expression of *B. turicatae* genes to *B. burgdorferi* genes. The expression of FlaB, BosR, and NapA from a set of three biological replicates for *B. burgdorferi* and *B. turicatae* were compared by immunoblot (b). The relative fold change in FlaB, BosR, and NapA protein levels in *B. burgdorferi* and *B. turicatae* samples were determined by densitometry (c). Data represent the result of three to six biological replicates ± *SD*. Statistical analysis was performed using an unpaired Student's *t*‐test. *p < 0.05 comparing expression of *B. burgdorferi* to *B. turicatae*

*B. burgdorferi* encodes a small repertoire of antioxidant defences that include coenzyme A disulfide reductase (*cdr*), neutrophil activating protein (*napA/dps/bicA*), a manganese‐dependent superoxide dismutase (*sodA*), thioredoxin (*trxA*), and thioredoxin reductase (*trxB*). Of these, only *cdr* and *sodA* have been characterised for their roles in detoxifying ROS (Boylan et al., 2006; Esteve‐Gassent et al., 2009). The expression of *cdr* and *napA* appear to be partly dependent on the *Borrelia* oxidative stress regulator (*bosR*) in *B. burgdorferi* (Boylan et al., 2006; Boylan, Posey, & Gherardini, 2003). The *B. turicatae* genome encodes homologues to each of these genes. Therefore, to determine whether the disparate sensitivities of *B. burgdorferi* and *B. turicatae* to ROS and RNS were the result of altered expression of these conserved antioxidant defence genes, we compared their expression in strains grown in mBSK medium by RT‐qPCR (Figure 6). The expression of *bosR* (3.8‐fold), *napA* (52.8‐fold), and *trxA* (8.6‐fold) were all lower in *B. turicatae* compared with *B. burgdorferi*, whereas the expression of *cdr* and *sodA* were similar in both strains. In contrast, *trxB* (6.5‐fold) was expressed at higher levels in *B. turicatae* compared with *B. burgdorferi*. This data suggests differences in the expression of several antioxidant defence genes may contribute to the varying sensitivities of *B. burgdorferi* and *B. turicatae* to killing by ROS and RNS.

Next, we investigated whether the disparate levels of *bosR* and *napA* transcripts in *B. burgdorferi* and *B. turicatae* resulted in differences in BosR and NapA protein levels. BosR and NapA levels were determined by Western blot. Densitometric analysis revealed comparable levels of BosR and NapA in *B. turicatae* and *B. burgdorferi* samples (Figure 3b,c). FlaB was used as a loading control and also showed similar levels of expression in both *B. turicatae* and *B. burgdorferi* samples.

## 3 DISCUSSION

RF *Borrelia* colonise and persist within salivary glands and midguts of *Ornithodoros* ticks (Dutton, Todd, & Newstead, 1905; Burgdorfer, 1951; Schwan & Hinnebusch, 1998; Schwan & Piesman, 2002; Boyle et al., 2014; Krishnavajhala et al., 2017). Transcriptomic and proteomic studies have provided insight into the potential differences in the microenvironments of the salivary glands and midguts that RF *Borrelia* occupy during colonisation of *Ornithodoros* species. RF *Borrelia* likely experience shifts in nutrient availability, osmolarity, temperature, pH, and exposure to effectors of the innate immune system, which include ROS and RNS. In this study, we showed the salivary glands of *O. turicata* ticks represent a highly oxidative environment, whereas midguts of uninfected ticks appear to present a primarily nitrosative environment. Therefore, the populations of *B. turicatae* present in the salivary glands and midguts of *O. turicata* ticks must withstand and adapt to distinct microenvironments. This is especially intriguing for the spirochetes colonising the *O. turicata* salivary glands, which appear to be preadapted for infection of mammalian hosts (Wilder et al., 2016). Our study suggests that ROS and RNS generated in the *O. turicata* salivary glands may serve as important environmental signals sensed by *B. turicatae* to prime it for mammalian infection. This possibility will require additional examination of (a) the impact of ROS and RNS on *B. turicatae* gene expression, (b) the molecular targets of ROS and RNS in *B. turicatae*, and (c) the impact of gene silencing of ROS/RNS‐producing enzymes or tick‐borne antioxidant defences on *B. turicatae* colonisation and vector–host transmission.

Our transcriptomic analyses of the *O. turicata* salivary glands revealed a variety of genes expressed that are related to ROS/RNS production or antioxidant defences. The most highly expressed genes involved in antioxidant defences in the salivary glands in our analyses encode two putative glutathione peroxidases (*gpx*), whose high levels of expression were confirmed by RT‐ddPCR (Figure 1a). Glutathione peroxidases (GPx) are antioxidant enzymes that typically use glutathione (GSH) to reduce H_2_O_2_ to H_2_O (Lubos, Loscalzo, & Handy, 2011). GPx enzymes also contribute to the detoxification of RNS, including both ONOO^−^ and *S*‐nitrosoglutathione (GSNO; Benhar, 2018). The observation of high levels of *gpx* expression, along with high levels of fluorescence in salivary glands incubated with DCF‐DA, indicates substantial levels of ONOO^−^ are formed in these tissues. Our transcriptomic analyses also suggest roles for a variety of superoxide dismutases, thioredoxin peroxidases, and catalase in limiting the oxidative and nitrosative stresses in *O. turicata* salivary glands. However, it is unclear if these antioxidant defences would benefit *B. turicatae* during colonisation of the salivary glands, as these enzymes are likely localised in the cytoplasm or the mitochondria of cells comprising the salivary gland acini.

In contrast to what we observed in the salivary glands, the expression of *gpx* was ~50‐fold lower in the *O. turicata* midgut (Figure 1). Surprisingly, the other antioxidant defence genes we examined were all expressed at higher levels in the midgut compared with the salivary glands. Thioredoxin peroxidase (Tpx) plays a major role in limiting nitrosative stress through the reduction of GSNO to GSH using the reducing power of thioredoxin (Benhar, 2018). The expression of *tpx* was ~10‐fold higher in the midgut compared with the salivary glands of unfed ticks (Figure 1). The elevated expression of *tpx* corresponds with the high levels of immunofluorescence for NOS (Figure 3) and the detection of RNS using DAF2‐DA (Figure 4), suggesting the midgut of unfed *O. turicata* ticks is a highly nitrosative environment.

DUOX enzymes have been implicated in the production of ROS in a variety of hematophagous arthropods, including *I. scapularis*, where DUOX‐generated ROS catalyse the formation of dityrosine complexes which dampen the immune response by limiting NO production from NOS (Yang et al., 2014). Our RNAseq analyses identified two *duox* genes (*duox1* and *duox2*). Interestingly, the expression of *duox1* was significantly increased in the salivary glands and midguts of *O. turicata* ticks infected with *B. turicatae*, suggesting *B. turicatae* may stimulate immune responses in the tick, specifically the production of ROS. This is similar to what has been observed in *I. scapularis* ticks where infection with *B. burgdorferi* stimulated increased expression of *duox* (Yang et al., 2014). The increased expression of *duox1* in *B. turicatae*‐infected *O. turicata* nymphs may also be associated with the formation of dityrosine complexes that help to dampen the tick immune response, as was observed in *I. scapularis* ticks. Overall, the expression of both *duox1* and *duox2* was higher in the midguts compared with the salivary glands of both uninfected and infected *O. turicata* ticks. Despite the higher levels of *duox1* and *duox2* expression, the increased levels of NOS detected by immunofluorescence (Figure 3), and the intense staining for RNS using the DAF2‐DA probe (Figure 4), the midgut is likely a site of nitrosative (rather than oxidative stress) in unfed ticks.

This study represents the first effort to directly compare the susceptibility of the RF spirochete *B. turicatae* and the LD spirochete *B. burgdorferi* to killing by ROS and RNS produced in vitro. The ability of *B. turicatae* to survive concentrations of H_2_O_2_ that were lethal to *B. burgdorferi* suggests it harbours antioxidant defences that are either absent in or not expressed in *B. burgdorferi*. A previous study indirectly suggested a serine protease (BhpA) in the antioxidant defences of the RF spirochete *Borrelia hermsii* (Guyard et al., 2006). BLAST analysis indicated that *bhpA* is conserved in RF *Borrelia*, whereas absent in LD spirochetes. Rather than directly assessing the role of BhpA in the resistance of *B. hermsii* to ROS, *B. hermsii bhpA* was inserted into *B. burgdorferi* and ROS killing evaluated. Transformed *B. burgdorferi* displayed enhanced resistance to killing by ROS produced in vitro or by human neutrophils. With the development of strategies to genetically manipulate *B. turicatae* (Lopez et al., 2013)*,* future studies will evaluate the role of BhpA in the spirochete's hyperresistance to H_2_O_2_.

The antioxidant arsenals of both RF and LD *Borrelia* appear to be somewhat limited. In this study, we examined the expression of several of these genes by RT‐qPCR. The expression of *cdr* and *sodA* were similar in both *B. turicatae* and *B. burgdorferi*, suggesting they are not involved in the disparate sensitivities of these bacteria to killing by ROS and RNS. In contrast, we observed the expression of *napA* was 50‐fold higher in *B. burgdorferi* compared with *B. turicatae* (Figure 6). The role of NapA (BicA/Dps) in the resistance of *B. burgdorferi* to ROS has been the subject of several studies. Recently, NapA has been described as a copper and iron binding protein, which acts as a metallothionein sequestering copper to prevent cytotoxicity (Wang et al., 2012). Moreover, a *napA*‐deficient *B. burgdorferi* strain was more resistant to killing by H_2_O_2_, suggesting an inverse relationship of *napA* expression and resistance to ROS (Li et al., 2007; Wang et al., 2012). Despite the observed differences in *napA* mRNA levels in *B. burgdorferi* and *B. turicatae*, NapA protein levels were comparable in both strains (Figure 6). Therefore, the differential susceptibilities of *B. burgdorferi* and *B. turicatae* to killing by ROS and RNS is not likely the result of differences in NapA levels. Thioredoxin (TrxA) and thioredoxin reductase (TrxB) are presumed to play an integral role in the antioxidant defences of both RF and LD *Borrelia* by maintaining the redox status of cysteine thiols subject to oxidation and/or *S*‐nitrosylation by ROS and RNS. Although we did see differences in the expression of *trxA* and *trxB* in *B. turicatae* and *B. burgdorferi*, it is unclear if this accounts for the differences in the ability of each strain to survive the challenge with ROS and RNS.

The data presented here support the hypothesis that RF *Borrelia,* including *B. turicatae,* are uniquely adapted to persist in the highly oxidative environment of *Ornithodoros* salivary glands. Although other RF *Borrelia* species transmitted by soft ticks will likely exhibit levels of resistance to ROS and RNS similar to those described here, it is unclear if this is true of RF spirochetes that are transmitted by hard‐bodied ticks (Armstrong et al., 1996; Barbour, Maupin, Teltow, Carter, & Piesman, 1996; Bunikis et al., 2004; Fraenkel, Garpmo, & Berglund, 2002; Fukunaga et al., 1995; Mun, Eisen, Eisen, & Lane, 2006; Scoles, Papero, Beati, & Fish, 2001) ‐ for example, *Borrelia miyamotoi* and *Borrelia lonestari* are vectored by *Ixodes spp*. and *Amblyomma americanum,* respectively. To date, only a single RF *Borrelia* species related to *B. miyamotoi* and *B. lonestari* has been reported to colonise the salivary glands of *Amblyomma geomydae* (Takano et al., 2012). Our findings provide evidence that the ability of vector‐borne pathogens, such as *B. turicatae*, to colonise the tissues of their arthropod vectors may be due in part to their ability to adapt to and withstand the ROS and RNS produced in various tissues, including the salivary glands and midguts.

## 4 EXPERIMENTAL PROCEDURES

### 4.1 Bacterial strains

*B. burgdorferi* strain B31 A3 (passage 2) and *B. turicatae* 91E135 isolate (passage 10) were grown under microaerobic conditions (5% CO_2_ and 3% O_2_) at 34°C in modified Barbour–Stoenner–Kelley (mBSK) medium throughout this study (Barbour, 1984; Battisti, Raffel, & Schwan, 2008).

### 4.2 *O. turicata* dissections, mRNA isolation, and sequencing

*O. turicata* used in the study was maintained in colony at Baylor College of Medicine and originated from field‐collected ticks in Kansas. Salivary gland sets from 25 uninfected ticks were dissected and placed into RNALater (ThermoFisher Scientific, Grand Island, NY). Samples were homogenised by multiple passages through a sterile 18G needle attached to a 1 ml syringe. Messenger RNA was isolated using FastTrack® MAG mRNA Isolation Kits (ThermoFisher), and the samples were evaluated on a Bioanalyzer 2100 (Agilent Genomics, Santa Clara, CA). Samples were sent to the North Carolina State Genomic Sciences Laboratory for Illumina RNA library construction and sequenced in an Illumina HiSeq 2500 DNA sequencer, utilising 125 bp single end sequencing flow cell.

### 4.3 Bioinformatic analyses

Custom bioinformatic analysis were describe elsewhere (Karim, Singh, & Ribeiro, 2011; Ribeiro, Slovak, & Francischetti, 2017) with modifications. Low‐quality reads were trimmed from Fastq files (less than 20) and contaminating adapter primer sequences were removed. *De novo* assembly of reads was performed using Abyss (with k parameters from 21 to 91 in 10‐fold increments) and SOAPde novo‐Trans (Birol et al., 2009; Xie et al., 2014). The combined FASTA files were further assembled using an iterative BLAST and CAP3 pipeline, as previously described (Karim et al., 2011). Coding sequences were predicted based on the presence of a signal peptide in the longer open reading frame by similarity to proteins in the Refseq invertebrate database from NCBI, and from proteins from Diptera deposited at NCBI's Genbank and from SwissProt. Reads for each library were mapped for the putative coding sequences using blastn with a word size of 25, 1 gap allowed, and 95% identity or higher was required. Up to five matches were allowed if the scores were identical to the largest score. Reads and values of reads per kilobase of transcript per million mapped reads (RPKM; Roberts, Trapnell, Donaghey, Rinn, & Pachter, 2011) for each coding sequence were mapped to an Excel spreadsheet. Automated annotation of proteins was based on a vocabulary of nearly 350 words found in matches to databases including Swissprot, Gene Ontology, KOG, Pfam, and SMART, Refseq‐invertebrates, and the acari subset of the GenBank sequences obtained by querying acari (organism) and retrieving all protein sequences. Raw reads were deposited on the Sequence Read Archive of NCBI under bioproject ID PRJNA270484. This Transcriptome Shotgun Assembly project has been deposited at the DDBJ/EMBL/GenBank under the accession GCJJ01000001‐GCJJ010065871.

### 4.4 Gene expression analyses using droplet digital PCR

Pools of midguts and salivary gland from five second nymphal stage ticks were dissected and individually pooled in 100 μl of RNAlater (ThermoFisher) and stored at −80°C. Tick tissues were pelleted by centrifugation and the RNAlater was removed. Tissue samples were transferred to 2 ml screwcap microcentrifuge tubes with 500–700 μl of RNAzol (Molecular Research Center, Cincinnati, OH). Pooled tissues were homogenised with a Bead Bug homogeniser (MIDSCI, St. Louis, MO) using 3.0 mm zirconium beads for 90 s. RNA was extracted from the RNAzol solution containing the homogenised tissue, and contaminating DNA removed following DNase treatment using Directzol columns (Zymo Research, Irvine, CA) per the manufacturer's instructions. RNA concentrations were measured using a TAKE3 plate and a Cytation 5 multi‐mode plate reader (Biotek, Winooski, VT). Following RNA extraction, cDNA was synthesised from 500 ng total RNA using the High Capacity cDNA Reverse Transcription kit (Applied Biosystems) per the manufacturer's instructions. Droplet digital PCR (ddPCR) was performed by adding 4 μl of cDNA into a PCR reaction containing 10 μl of 2x ddPCR master mix for probes (Bio‐Rad, Hercules, CA), 1 μl of 20x PrimeTime mini qPCR assay probe set (IDT, Coralville, IA; Table 2), and 5 μl molecular grade H_2_O. Droplets were created with the Droplet Generation Oil for Probes in the QX200 droplet generator (Bio‐Rad). Droplets were then transferred to a 96 well plate and PCR amplified (94°C for 30 s and 60°C for 60 s for 40 cycles) using the C1000 thermocycler. The fluorescence signal within droplets were detected by the QX200 droplet reader and data were analysed by Quantasoft Analysis Pro.

**Table 2:** Oligonucleotides for RT‐qPCR and ddPCR

Gene Target	Primer	Sequence (5′ ≥ 3′)
*sodA*_*Bt*_	F	TTGGGCTTGGCTAGTTCTTC
	R	AGCATGTTCCCAAACATCAATAC
*bosR*_*Bt*_	F	GTCGGAATTACTAATGACCCTATCT
	R	TTTGGATTTGAGGCAATGTGTAG
*napA*_*Bt*_	F	CATCTGATTATGGGACGGCTAAT
	R	TGGACACTCACAATCACTTAACA
*cdr*_*Bt*_	F	TGGAGCTTGTGGATTACCATATT
	R	TTCATCAGGTGTTCTGGCTATC
*trxB*_*Bt*_	F	GGCTTACGGCTGGAATCTATAC
	R	ACTTCCGTAGTCGTTGTAAGC
*trxA*_*Bt*_	F	GCTTGCTCCGATTTATGATGAAC
	R	GAACACCAAGCGCCATAGA
*flaB*_*Bt*_	F	TGCTGTGCAGTCTGGTAATG
	R	GCATTTGGTTGTATTGAGCTTGA
*sodA*_*Bb*_	F	GTGTCCTGAGAGTGGCCTTA
	R	GTAATAGGCATGCTCCCAAA
*bosR*_*Bb*_	F	CCAAGCCTATCAAAAGCAA
	R	TAGGGTGGACTTGATTGCAT
*napA*_*Bb*_	F	GGAGCTTGATATTGAATCAACTT
	R	CTTCAAAATCTCAGTAAGACTGC
*cdr*_*Bb*_	F	TGGAGCTTGTGGATTACCATATT
	R	TTCATCAGGTGTTCTGGCTATC
*trxB*_*Bb*_	F	TGTTGGCTATAAGCCAAATACAGA
	R	CAGATGCAATAAACCCCTCA
*trxA*_*Bb*_	F	GAGGTGATAGACCCGCAATA
	R	TGTTCCTTATCTGTATCTACTTTG
*flaB*_*Bb*_	F	AGCTCCCTCACCAGAGAAA
	R	GCATCACTTTCAGGGTCTCA
*gpx*	Hyb*	AGGGAAAGCCAGTGAAGCGATACC
	F	ATCGACTGGAACGCATTGT
	R	ACGACCCTCAACACTGTAATG
*tpx*	Hyb*	TGTGGAAGAAACACTTCGCCTCGT
	F	GGGCACACTTCTCCATTCTT
	R	GCAACGTCTGCGACAGATAA
*sod‐1*	Hyb*	AGTCTGGACGCACATTTCTGTACTGC
	F	GCCTTTCCGCAATGTTGTTC
	R	TGGGAACACGCCTACTATCT
*sod‐2*	Hyb*	AGGGACTTCACGGATTTCACGTTCAC
	F	TCCGTGTGGGTTGAAATGG
	R	GGCGGTTACCGGTGAAATAA
*cat*	Hyb*	TTCAGATGGGTGGCGACGTTTGTC
	F	GTGGCACGAGTCCTGATAAA
	R	GGAAGATGCTGTCTGAAGAAGA
*nos*	Hyb*	AGAGCACGGGAAAGGAAAGAAGCA
	F	CTCACAAAGCAGGCCATTTC
	R	CGTCGCTGTTGTCACCTATAA
*duox1*	Hyb*	CCATCAACATCCAGAGAGGACGCG
	F	GGCAGTCTTATAGTCAGGAATGG
	R	GATCTTCGAGGCCGTGTATTT
*duox2*	Hyb*	TGCTTGGAACTCGTGGCTGATACC
	F	GGAATGGACGTATGCGAGAA
	R	GTCACAGAATACACAGGCTACA

* 20x PrimeTime qPCR Probe Assays include the forward and reverse primers, along with a hybridization oligo labelled with the fluorescent dye 6‐carboxyfluorescein (6‐FAMTM), an internal ZEN Quencher, and Black Hole QuencherTM 1 (BHQTM‐1).

### 4.5 Immunofluorescent detection NOS in tick salivary glands and midguts

Salivary glands and midguts harvested unfed *O. turicata* nymphs, were fixed and permeabilized using the Fix and Perm Kit (ThermoFisher) per the manufacturer's instructions. Tick tissues were incubated in PBS in the presence of BSA (10 mg ml^−1^) and rabbit serum as a negative control or with a 1:100 dilution of a universal NOS antibody (ThermoFisher) for 3 h. Tissues were then subjected to 3 × 0.5 ml washes in PBS and then incubated with a 1:200 dilution of the Goat anti‐Rabbit IgG secondary antibody Alexa Fluor 488 conjugate (ThermoFisher) and a 1:400 dilution of DAPI (ThermoFisher) for an additional 3 h at RT. Tissues were washed as described above and then visualised by fluorescent microscopy using the Blue channel (Ex_λ_ = 355 nm, Em_λ_ = 433 nm) and the Green channel (Ex_λ_ = 480 nm, Em_λ_ = 517 nm) of the ZOE Flourescent Cell Imager (Bio‐Rad).

### 4.6 Detection of ROS and RNS in tick salivary glands and midguts

ROS and RNS generated in *O. turicata* salivary glands and midguts were detected with the fluorescent probes 2,7‐dichlorodihydrofluorescein diacetate (DCF‐DA) and 4,5‐diaminofluorescein diacetate (DAF‐2), purchased from Cayman Chemical (Ann Arbor, MI). DCFH is readily oxidised by ONOO^−^ to the highly fluorescent product dichlorofluorescein (DHF). DAF‐2 reacts with NO in the presence of oxygen to produce triazolofluorescein (DAF‐2 T). Briefly, salivary glands and midguts dissected from stage 2 *O. turicata* nymphs were rinsed extensively in PBS, and transferred to individual 1.5 ml microcentrifuge tubes with 25 μM DCF‐DA or DAF‐2 for 10 min at RT. Following incubation, tick tissues were placed in individual pools of PBS on glass microscope slides, and imaged using the brightfield and Green channel (Ex_λ_ = 480 nm, Em_λ_ = 517 nm) of the ZOE Flourescent Cell Imager (Bio‐Rad).

### 4.7 ROS and RNS susceptibility assays

*B. burgdorferi* and *B. turicatae* strains were grown to late log‐phase (~5 × 10^7^ cells ml^−1^) under microaerobic conditions (5% CO_2_, 3% O_2_) at 34°C in pyruvate‐free mBSK media. One milliliter aliquots of cells were transferred to 5 ml polypropylene culture tubes (MIDSCI) and incubated at 34°C under microaerobic conditions for 2 h in the presence or absence of either H_2_O_2_ (Sigma, St. Louis, MO) or diethylamine NONOate (Cayman Chemical). Following treatment, serial dilutions of cells were prepared in mBSK and were plated on semi‐solid mBSK media as previously described (Raffel, Williamson, Schwan, & Gherardini, 2018). Plates were incubated at 34°C under microaerobic conditions for 7–14 days, and colony forming units (CFUs) were enumerated. Percent survival was determined by dividing CFUs from the 2 h time point samples by the CFUs from the 0 h time point.

### 4.8 RNA isolation and RT‐qPCR analysis

Cultures of *B. burgdorferi* and *B. turicatae* were grown in 12 ml mBSK media to a cell density of ~5 × 10^7^ cells ml^−1^ under microaerobic conditions (5% CO_2_, 3% O_2_), pelleted by centrifugation at 3,000 xg, and cell pellets resuspended in 1 ml RNAzol (Molecular Research Center). Total RNA was extracted from the cell lysates per the manufacturer's instructions. RNA was resuspended in 50 μl H_2_O, and RNA concentrations were measured using a TAKE3 plate and a Cytation 5 multi‐mode plate reader (Biotek). Following RNA extraction, cDNA was synthesised from 1 μg total RNA using the High‐Capacity cDNA Reverse Transcription Kit (ThermoFisher) per the manufacturer's instructions. Quantitative reverse transcription PCR (RT‐qPCR) reactions were prepared for cDNA samples using the Bullseye EvaGreen and TaqProbe qPCR master mixes (MIDSCI) and oligonucleotide primers listed in Table 2. The RT‐qPCR reactions were performed using a CFX Connect Real‐time PCR Detection System (Bio‐ Rad) with cycling conditions of 95°C for 10 s, 59°C for 20 s, and 72°C for 30 s followed by melt‐curve analysis to determine the efficiency and specificity of the qPCR reactions. The efficiency of each primer set was determined using CFX Manager software (Bio‐Rad) by performing qPCR on 10‐fold serial dilutions of purified *B. burgdorferi* or *B. turicatae* DNA. Primer efficiencies for each primer set ranged from 95% to 100%. The relative expression of selected genes normalised to the flagellin gene (*flaB*) was determined using the 2^−ΔΔ*CT*^ method (Livak & Schmittgen, 2001). The Cq values for *flaB* showed a coefficient of variance of less than 3% for both *B. burgdorferi* and *B. turicatae* cDNA samples.

### 4.9 SDS‐PAGE and immunoblot

Whole cell lysates were prepared from 30 ml cultures of *B. burgdorferi* and *B. turicatae* after growth to a cell density of 2 × 10^8^ cells ml^−1^ in mBSK. Cultures were centrifuged at 3,200 g for 17 min to pellet spirochetes. Pellets were washed twice with HN Buffer (10 mM HEPES and 10 mM NaCl at pH 8.0), and whole cell lysates were prepared in Lysis Buffer (4% SDS and 0.1 M Tris, pH 8.0). Protein content was normalised using a BCA. Protein content was normalized using a BCA protein assay per the manufacturer's instructions (Thermo Fisher Scientific, Waltham, MA). Samples were run on SDS‐PAGE using a Mini‐Tetra system (Bio‐Rad, Hercules, CA), and protein bands were visualized using the EZstain system in conjunction with the Gel Doc EZ Imager (Bio‐Rad). Membranes were blocked in 5% milk in TBST for 2 h and then washed in TBST. Membranes were then incubated with primary antibodies, including α‐FlaB (Thermo Fisher Scientific, Waltham, MA) at a dilution of 1:4000, α‐BosR (courtesy of Dr. Frank Gherardini, RML) at a dilution of 1:500, and α‐NapA (courtesy of Dr. Frank Gherardini, RML) at a dilution of 1:500. Binding of primary antibodies was detected using an HRP‐conjugated secondary antibody, and blots were developed using the ECL chemiluminescence substrate (LI‐COR, Lincoln, NE). Blot imaging was performed on the ChemiDoc Imaging system (Bio‐Rad, Hercules, CA). Immunoblots were quantified and evaluated for relative fold change using the Image J software. A value of “1” was assigned to *B. burgdorferi* to determine the relative fold change between *B. burgdorferi* and *B. turicatae*.

### 4.10 Statistical analysis

Data are presented as mean ± standard deviation (*SD*). To determine statistical significance between multiple comparisons, two‐way analysis of variance were performed, followed by a Tukey's post‐test. A two‐tailed, student's *t*‐test with the Holm–Sidak method was used to determine statistical significance between two groups. Data were considered statistically significant when *p* < 0.05.

## CONFLICT OF INTEREST

The authors declare that they have no conflicts of interest.

## AUTHOR CONTRIBUTIONS STATEMENT

T. B., A. Z., and J. L. wrote the main manuscript text. T. B., A. Z., W. B., L. O., J. V., and J. L. performed the experiments. T. B., A. Z., W. B., and J. L. prepared the figures and tables. All authors reviewed the manuscript.

## Supporting information

Data S1 Supporting information
