# Supplementary material for: The relapsing fever spirochete Borrelia turicatae persists in the highly oxidative environment of its soft‐bodied tick vector
Source: Cell Microbiol. 2019 Jan 4;21(2):e12987. doi: 10.1111/cmi.12987 (PMC6454574; doi:10.1111/cmi.12987)
Supplement: Supplementary file 1 — Data S1 Supporting information [file CMI-21-na-s001.docx]

| **Class** | **Total families** | **RPKM** | **Reads per families** | **% abundance** |
| --- | --- | --- | --- | --- |
| **Secreted protein** | 2138 | 410940 | 192.21 | 28.89877482 |
| **Immunity** | 154 | 11402 | 74.04 | 0.801829538 |
| **Cytoskeletal proteins** | 280 | 51097 | 182.49 | 3.593324323 |
| **Detoxification** | 105 | 4137 | 39.40 | 0.290928679 |
| **Oxidant metabolism/Detoxification** | 56 | 5395 | 96.34 | 0.379395752 |
| **Extracellular matrix** | 195 | 19982 | 102.47 | 1.405205914 |
| **Amino acid metabolism** | 125 | 13075 | 104.60 | 0.919480899 |
| **Carbohydrate metabolism** | 225 | 8789 | 39.062 | 0.618074006 |
| **Energy metabolism** | 199 | 20251 | 101.76 | 1.424122959 |
| **Intermediary metabolism** | 57 | 1846 | 32.39 | 0.129817342 |
| **Lipid metabolism** | 285 | 12734 | 44.68 | 0.895500556 |
| **Nucleotide metabolism** | 160 | 5444 | 34.03 | 0.382841607 |
| **Nuclear export** | 45 | 5080 | 112.89 | 0.357243822 |
| **Nuclear regulation** | 319 | 13914 | 43.62 | 0.978482389 |
| **Protein export** | 392 | 32075 | 81.82 | 2.255629052 |
| **Protein modification** | 369 | 35799 | 97.02 | 2.517514089 |
| **Proteasome machinery** | 279 | 28100 | 100.72 | 1.976092793 |
| **Protein synthesis machinery** | 340 | 258269 | 759.61 | 18.16240248 |
| **Signal transduction** | 1246 | 67898 | 54.50 | 4.774830907 |
| **Storage** | 13 | 5375 | 413.46 | 0.37798928 |
| **Transposable element** | 277 | 5927 | 21.40 | 0.4168079 |
| **Transcription factor** | 185 | 12452 | 67.31 | 0.875669305 |
| **Transcription machinery** | 798 | 116265 | 145.70 | 8.176171837 |
| **Transporters and channels** | 405 | 37629 | 92.91 | 2.646206253 |
| **Unknown conserved** | 1172 | 110598 | 94.37 | 7.77764807 |
| **Unknown conserved membrane protein** | 305 | 28598 | 93.76 | 2.01111394 |
| **Unknown product** | 825 | 89379 | 108.34 | 6.285451878 |
| **Viral product** | 40 | 9548 | 238.70 | 0.671449608 |
|  |  |  |  |  |
| **Total** | 10,989 | 1,421,998 |  |  |
